# The Role of Preoperative Urinalysis in Predicting Postoperative Infection After Retrograde Intrarenal Surgery in Patients with Sterile Urine Culture

**DOI:** 10.5152/tud.2022.21331

**Published:** 2022-03-01

**Authors:** Ozgur Kazan, Mehmet Caglar Cakici, Ferhat Keser, Meftun Culpan, Ozgur Efiloglu, Asif Yildirim, Gokhan Atis

**Affiliations:** 1 Department of Urology, Kocaeli State Hospital, Kocaeli, Turkey; 2 Department of Urology, İstanbul Medeniyet University Faculty of Medicine, İstanbul, Turkey

**Keywords:** Leukocyte esterase, nitrite, pyuria, retrograde intrarenal surgery, urolithiasis

## Abstract

**Objective:**

Postoperative urinary tract infection is the most common complication of retrograde intrarenal surgery, and no consensus has been obtained that would reveal exact reasons yet. It was aimed to determine the possible factors, especially preoperative urinalysis, of postoperative urinary tract infection after retrograde intrarenal surgery.

**Material and Methods:**

Patients who underwent retrograde intrarenal surgery in our clinic between 2013 and 2019 were retrospectively screened. Stone size <1 cm and >2 cm and pediatric patients were excluded from the study. The patients were divided into 2 groups as those with and without urinary infections in the early postoperative period. Urine analysis parameters and sterile urine cultures that were taken before the procedure were also analyzed separately.

**Results:**

A total of 289 patients meeting the defined criteria were included in the study. There was no statistical difference between the 2 groups in terms of demographics. The number of patients with previous urinary tract infection history (55% vs. 20.5%) and operation time (62.5 ± 16.6 minutes vs. 60 ± 19.4 minutes) were significantly higher in those who had postoperative early urinary tract infection. Among urinalysis, the presence of pyuria, leukocyte count, leukocyte esterase positivity, and nitrite positivity were significantly higher in those who had postoperative early urinary tract infection. In multivariate analysis, urinary tract infection history, operation time, and nitrite positivity were found as independent factors in predicting postoperative early urinary tract infection.

**Conclusion:**

Previous urinary tract infection history, prolonged operation time, and nitrite positivity in urinalysis were determined as independent risk factors for postoperative urinary tract infection in kidney stones between 1 and 2 cm.

## Main Points

- This study focused on the predicting role of an overlooked and simple test such as “urinalysis” on postoperative urinary tract infection (UTI) after retrograde intrarenal surgery (RIRS).
- Prolonged operation time, UTI history, and preoperative nitrite positivity in urinalysis are independent risk factors for postoperative UTI after RIRS.
- Sterile urine culture does not exclude postoperative UTI and nitrite positivity in preoperative urinalysis should be considered.

## Introduction

Endourological interventions such as retrograde intrarenal surgery (RIRS), percutaneous nephrolithotomy, and shock wave lithotripsy are the most common treatment methods for kidney stones between 1 cm and 2 cm in size. The European Association of Urology and American Urological Association (AUA)/Endourological Society guidelines also suggest these treatment options.^1,2^ Retrograde intrarenal surgery succeeds with miniaturized flexible ureteroscopes as a minimally invasive operation with limited complications. Retrograde intrarenal surgery has a complication rate between 9% and 25%.^2,3^ Urinary tract infection (UTI) is the most frequent one after RIRS.^4,5^ De la Rosette et al^6^ analyzed 11 885 patients for the type of complications associated with ureteroscopic lithotripsy, and they reported the most frequent complication was postoperative fever (1.8%) followed by hematuria requiring blood transfusion (0.2%). The majority of complications were Clavien grade 1 or 2.

Since postoperative infection is the most common complication, prophylactic empirical antibiotic use has become widespread and is now used as a standard to prevent postoperative UTI. Lo et al^7^ showed that prophylactic empirical antibiotics reduce the rate of bacteriuria, but without clinical impact. Because of the empirical use of antibiotics, increasing antimicrobial resistance, and unpredictable patient factors, the rate of postoperative UTI is still high and severe consequences such as urosepsis are seen in daily practice.

Several studies defined the predictive factors of postoperative UTI. Antimicrobial resistance, prolonged operation time, preoperative pyuria, the stone itself, diabetes mellitus (DM), obesity, and urinary catheters are some of the most blamed factors for postoperative UTI.^8,9^ However, there is no consensus on the factors and how to prevent postoperative infection.

Our study aimed to investigate the factors causing urinary tract infection after RIRS to decrease the rate of procedure-related complications. In addition to patient- and procedure-related factors, preoperative simple urine characteristics are especially examined.

## Material and Methods

From September 2013 to July 2019, medical records of 642 patients, who underwent RIRS for proximal ureteral and/or kidney stones, were reviewed retrospectively. A total of 289 patients with a total stone size between 1 cm and 2 cm were included in the study. To create a homogeneous study population in terms of treatment indication according to guidelines and possible complications, only stones in the upper ureter and renal stones between 10 mm and 20 mm in size were included. In total, 124 patients whose stones were smaller than 10 mm, 217 patients whose stones were larger than 20 mm, and 17 patients with an indwelling urinary catheter or who were doing clean intermittent catheterization were excluded.

The patients were divided into 2 groups, those with and without postoperative UTI. The following demographic data of the patients were retrospectively analyzed: age, sex, body mass index (BMI), accompanying DM, hypertension (HT), Charlson Comorbidity Index (CCI), stone diameter, and stone location; perioperative characteristics such as preoperative hydronephrosis, UTI history, defined as any symptomatic UTI with positive urine culture, and preoperative Double-J stent; operative features such as access sheath use, operation time, and residual fragments (fragments larger than 2 mm).

Postoperative UTI was defined as fever higher than 38.3°C and positive results in urine or blood bacterial culture within 1 month after ureteroscopy. Sterile urine cultures were obtained from all patients during the preoperative 30-day period. Urinalysis was also obtained from each patient at the same time with urine culture before the operation. Urinalysis values, such as count of leukocytes, presence of pyuria, leukocyte esterase, and nitrite positivity were analyzed. Pyuria was defined as ≥5 white blood cells/mm^3^ in urinalysis. Prophylactic antibiotics were chosen individually for each patient, according to former frequent positive urine cultures; if former urine culture positivity is not present, cefuroxime 1500 mg was chosen for prophylaxis. Patients who were treated with antibiotics were not included in this study. 7.5 F Storz Flex-X2 was used for flexible ureterorenoscope (URS); a hydrophilic-coated ureteral access sheath (9.5/11.5F or 12/14F) was preferred.

Statistical analyses were performed using Statistical Package for the Social Sciences Statistics ver. 22 software (IBM SPSS Corp.; Armonk, NY, USA). Independent samples *t*-test was used for comparing the means. Where the variables between groups were not normally distributed, the Mann–Whitney *U* test was preferred. Chi-square test or Fisher’s exact test was used for categorical variables. Logistic regression was performed for multivariate analysis and *P* value <.05 was considered as statistically significant.

Ethical approval for this study was granted by the Istanbul Medeniyet University Ethics Committee (No: 2021/0124).

## Results

Postoperative UTI was observed in 20 (6.9%) of the 289 patients who had RIRS with 1-2 cm renal and/or proximal ureteral stones. Twenty patients with postoperative UTI were defined as group 1, and 269 patients without postoperative UTI were defined as group 2. Most of the patients who developed postoperative UTI were females, whereas the rate of male patients was higher in the non-UTI patients (55% vs. 39.8%, *P* = .181). Age, BMI, accompanying DM, HT, and CCI scores were similar between the 2 groups. Median stone diameters were similar in both groups (median 13.5 mm vs. 15 mm, *P* = .285). Patients with postoperative UTI had a higher rate of UTI history (55% vs. 20.5%, *P* = 0.000) and longer operative times (median 62.5 ± 16.6 minutes vs. 60 ± 19.4 minutes, *P* = .008). Preoperative hydronephrosis, access sheath use, and residual fragments did not affect either of the 2 groups. Patients with postoperative UTI had a higher rate of preoperative Double-J stent, but this was also not statistically significant (40% vs. 23.4%, *P* = .097) (Table 1). 

To predict the postoperative UTI, the features of urinanalysis, which were obtained at the same time with sterile urine cultures, were analyzed. Patients with postoperative UTI had higher leukocyte count in urinanalysis (median 22.5 vs. 7.0, *P* = .009) and pyuria was more common in this group (85% vs. 59.8%, *P* = .026). Leukocyte esterase positivity was more common in patients with postoperative UTI (90% vs. 63.6%, *P* = .017) and they had higher rates of nitrite positivity (35% vs. 8.6%, *P* = .000) (Table 2). Besides sensitivity, specificity, positive predictive value, and negative predictive value, the diagnostic odds ratios (DOR) [sensitivity/(1 − sensitivity)/(1 − specificity)/specificity] were analyzed for each urine test parameters and their combinations. Nitrite alone has the highest DOR, whereas a combination of nitrite and leukocyte esterase has the same DOR. Pyuria and leukocyte esterase have high sensitivity (85% and 90%) but significantly low specificity rates (40.1% and 36.4%). Nitrite has low sensitivity (35%) and high specificity (91.4%), above all, nitrite has a higher positive predictive value than pyuria and leukocyte esterase (23.3% vs. 9.5%) (Table 3).

In multivariate analysis adjusting for covariates that were determined by univariate analysis such as UTI history, operation time, leukocyte count in urinanalysis, presence of pyuria, leukocyte esterase and nitrite positivity; UTI history, prolonged operative time, and nitrite positivity in preoperative urinanalysis were found to be independent risk factors for postoperative UTI in RIRS patients with stones between 1 and 2 cm (Table 4).

Stone analysis could not be performed in all patients. In group 1, 8 patients (40%) had stone analysis. The most frequent stone types in this group were calcium oxalate (including mixture with calcium phosphate) in 4 patients (50%), uric acid stone in 1 patient (5%), struvite stones in 2 patients (%10), and cystine stone in 1 patient (%5). In group 2, 82 patients (30.5%) had stone analysis, with the most frequent type being calcium oxalate (including mixture with calcium phosphate) in 63 patients (76.8%), 12 patients (%14.6) had uric acid stones, and 7 patients (8.6%) had cystine stone.

## Discussion

With the technological advances and miniaturization of the instruments, the most common treatment method in the proximal ureter and kidney stones between 1 cm and 2 cm is RIRS. The fact that it is a minimally invasive method means that is often preferred treatment although it has some complications, the most common in daily practice being postoperative UTI. De Coninck et al^10^ systematically reviewed and reported on the incidence of postoperative UTI after ureteroscopy at between 0.2% and 15%. Fourteen recent studies reported the rate of postoperative UTI and that various rates might be related to different characterizations of postoperative UTI and also different preoperative preparations and prophylaxis.

In our study, 20 (6.9%) of 289 patients had postoperative UTI after RIRS. Our postoperative infection rate seems to be lower than that in some studies, which may have been achieved by obtaining a sterile urine culture in all patients and following a regular and patient-based antibiotic prophylaxis strategy during the operation preparation phase. The fact that it seems to be at a higher rate than that in some studies may be because we are a referral center for stone disease with a high number of complex cases and some unpredictable factors. Which patients would be faced with postoperative UTIs has been frequently investigated in many previous studies. Some factors, such as female gender, DM, hydronephrosis, stone size, presence of a urethral catheter, ureteral stent, and percutaneous nephrostomy have been expressed mostly in predicting postoperative infection.^9,11,12^

Prolonged operation time is a well-known predictor of postoperative UTI after ureteroscopy. There is no consensus on optimal operation time or no time limit, but in order not to keep high intrarenal pressure for a long time, the general approach is for operation times to be as short as possible. Southern et al^13^ analyzed the risk factors of postoperative fever and systemic inflammatory response syndrome after 3298 URS procedures. Their postoperative infectious complications rate was 6.9%, and female gender, surgical time, CCI, and positive preoperative urine culture were independent predictors. Patients with postoperative infectious complications had an overall longer operative time (57 vs. 49 minutes). Moses et al^14^ defined the predictors of postoperative UTI after URS as operation time longer than 2 hours, pre-stenting, and non-compliance to AUA antibiotic prophylaxis guidelines. Kim et al^8^ found operation time as the only independent risk factor for postoperative UTI after URS (82.8 vs. 64.5 minutes) and defined 70 minutes as a cut-off value for operative time with a sensitivity of 58.1% and specificity of 61.7%. In our study, a longer operation time was also identified as an independent risk factor for postoperative UTI after RIRS in 1-2 cm ureteral/renal stones (median 62.5 ± 16.6 vs. 60 ± 19.4 mm; *P* = .006). However, our study differs from other studies in that, we obtained sterile preoperative urine culture before the procedure and had similar stone burdens. As previously mentioned, long operative time brings high pressure into the renal pelvis, which leads to systemic absorption of high irrigation volume.

Urinary tract infection history is a known risk factor for reinfection. Having such a preoperative history can also help in predicting that a UTI may occur in the postoperative period. Mitsuzuka et al^15^ found preoperative acute pyelonephritis as an independent predictor of postoperative UTI after URS. Their study reported that postoperative complication rates were higher in patients who were treated with antibiotics preoperatively. In contrast, those patients were excluded from our study, and a sterile urine culture was obtained from all patients within 1 month and prepared for the operation. Youssef et al^16^ emphasized that preoperative sepsis leads to higher postoperative UTI and sepsis rates after URS. In our study, we have defined the history of UTI as symptomatic UTI over the past year that was accompanied by positive urine culture. It was observed that postoperative infection developed significantly more frequently in the patient group with a history of UTI than in the patient group without (55% vs. 20.5%, *P* = .000).

We would like to emphasize that, in our study, the operation was performed after obtaining a sterile urine culture in the preoperative period in all patients. Obtaining a sterile urine culture is important, especially considering that these operations lead to high intrarenal pressure. Microbiome studies, which have been frequently mentioned, have also shown that the known sterile genitourinary system is not actually sterile. From the improvements in advanced polymerase chain reaction and 16S rRNA sequencing techniques, we have seen that many organisms colonize the urinary tract.^17^

Although obtaining a sterile urine culture is important, especially in stone patients, sterile urine culture can be misleading. A simple urine analysis is used in routine daily practice but is overlooked alongside the urine culture. In our study, we have seen that the urine analysis taken at the same time as sterile urine culture may provide us an insight into the development of infection in the postoperative period. The greatest advantage of urine analysis is that it is a simple method. Mitsuzuka et al^15^ observed that preoperative pyuria was associated with postoperative UTI and that postoperative UTI progressed more seriously as the level of pyuria increased. Kohada et al^18^ analyzed the risk factors for postoperative UTI after transurethral resection of bladder tumors and they have observed that asymptomatic pyuria was associated with postoperative UTI. Chen et al^19^ reported the predictors of postoperative UTI following percutaneous nephrolithotomy. They characterized pyuria, nitrite positivity, positive urine culture, and positive stone culture as predictors of postoperative UTI and urosepsis. The coexistence of pyuria and nitrite positivity was found to be the most valuable parameter for predicting postoperative infection, and 90% correlation was found with culture positivity. Fan et al^20^ identified the risk factors for developing postoperative uroseptic shock following percutaneous nephrolithotomy. Preoperative urine nitrite, stone size, and postoperative blood leukopenia were the independent risk factors. Contrary to our study, preoperative sterile urine culture was not obtained in any of the 4 studies that investigated preoperative urine analysis. In our study, we have observed that all the urine analysis parameters play a role in univariate analysis for predicting postoperative UTI, with nitrite positivity as the only independent predictor of postoperative UTI after RIRS (OR: 8.31, *P* = .000). Among the urinalysis parameters, nitrite has the highest specificity and negative predictive value. We showed that 246 of 259 patients (94.9%) with negative nitrite did not have postoperative UTI. Nitrite had the highest DOR (5.76) predicting postoperative UTI following RIRS, while its combination with other urine analysis parameters did not add value in predicting postoperative UTI.

The main strength of our study is identifying the urine analysis parameters that may help to predict postoperative infection, despite sterile urine culture. It is seen that in other studies, the condition of sterile urine culture was not sought. Our study is the first to cover this aspect. Especially in ureteral/renal stone patients, obtaining preoperative sterile urine culture is particularly important although sterile urine culture does not guarantee that there will be no infection in the postoperative period. In this study, we aimed to see these factors. 

Among the main limitations of our study are retrospective modality, limited stone analysis in all patients, and infection stone rates. Since our clinic focuses on complex cases in terms of stone diseases, its applicability to the general can also be seen as a limitation. The limited number of patients, especially in the postoperative infection group, may be regarded as a limitation. Although there is no consensus regarding the duration between preoperative sterile urine culture and the operation in current guidelines, 1 month may be a long interval. Although this period is less than 2 weeks in most of the patients included in the study and there is a similar time interval between the groups, this period should be kept as short as possible.

To conclude, nitrite positivity in preoperative urinanalysis, history of UTI, and prolonged operation time are the independent risk factors that may predict the postoperative infection in RIRS for ureteral/renal stones between 1 cm and 2 cm in size. Although preoperative sterile urine culture is important and should be obtained, a simple urine analysis, especially nitrite positivity should be also considered. A patient-based approach, considering risk factors, is recommended.

## Figures and Tables

**Table 1.** Perioperative and Demographic Features

	Postoperative UTI		*P*
	Yes (n = 20)	No (n = 269)	
Age (years) Ave ± SD	47.8 ± 12.6	47.9 ± 14.7	.988^‡^
Sex			
Male	9 (45%)	162 (60.2%)	
Female	11 (55%)	107 (39.8%)	.181^†^
BMI Med ± SD	26.3 ± 3.2	27.4 ± 4.7	.436^§^
DM			
Yes	3 (15%)	57 (21.1%)	
No	17 (85%)	212 (78.9%)	.510^†^
HT			
Yes	4 (20%)	69 (25.6%)	
No	16 (80%)	200 (74.4%)	.575^†^
Charlson comorbidity index			
0-1	14 (70%)	202 (77.9%)	
≥2	6 (30%)	57 (22.1%)	.410^†^
Stone diameter (mm) Med ± SD	13.5 ± 4.1	15 ± 3.6	.285^§^
Stone localization			
Upper calyx	3 (15%)	9 (3.3%)	
Middle calyx	2 (10%)	29 (10.8%)	
Lower calyx	4 (20%)	90 (33.4%)	
Renal pelvis	8 (40%)	72 (26.8%)	
Proximal ureter	0 (0%)	12 (4.5%)	
Multiple	3 (15%)	67 (24.9%)	.090^†^
Preoperative hydronephrosis			
Yes	8 (40%)	140 (52.1%)	
No	12 (60%)	128 (47.9%)	.550^†^
UTI history			
Yes	11 (55%)	55 (20.5%)	.001
No	9 (45%)	214 (79.5%)	**.000** ^**^
Preoperative Double-j stent			
Yes	8 (40%)	63 (23.4%)	
No	12 (60%)	206 (76.6%)	.097^†^
Access sheath use			
Yes	10 (50%)	157 (58.4%)	
No	10 (50%)	112 (41.6%)	.465^†^
Operation time (min) Med ± SD	62.5 ± 16.6	60 ± 19.4	**.008** ^**^
Residual fragment			
Yes	3 (30%)	93 (34.6%)	
No	17 (70%)	176 (65.4%)	.073^†^

UTI, urinary tract infection; Ave, average; Med, median; BMI, body mass index; DM, diabetes mellitus; HT, hypertension, SD Standard Deviation.

^†^Chi-square test, ^§^Mann–Whitney *U* test, ^‡^independent samples *t*-test.*: p < .05, **: p < .01.

**Table 2.** Relationship Between Preoperative Urine Analysis and Postoperative UTI

	Postoperative UTI		*P*
Preoperative Urine Analysis	Yes	No	
WBC Med ± SD	22.5 ± 196.2	7.0 ± 86.9	**.009** ^**^
Pyuria			
No	3 (15%)	108 (40.2%)	
Yes	17 (85%)	161 (59.8%)	**.026** ^*^
Leukocyte esterase			
−	2 (10%)	98 (36.4%)	
+	18 (90%)	171 (63.6%)	**.017** ^*^
Nitrite			
−	13 (65%)	246 (91.4%)	.001
+	7 (35%)	23 (8.6%)	**.000** ^**^

UTI, urinary tract infection; Med, median; WBC, white blood cells; Pyuria, ≥ 5 WBC/mm^3^ in urinalysis.

SD Standard deviation.

^†^Chi-square test, ^§^Mann–Whitney *U* test.*: p < .05, **: p < .01.

**Table 3.** Urine Analysis as a Predictor of Postoperative UTI

	Preoperative Urine Analysis for Prediction of Postoperative UTI						
	WBC+	LE +	N+	WBC+LE+	WBC+N+	LE+N+	WBC+LE+N+
Sensitivity	85%	90%	35%	85%	30%	35%	30%
Specificity	40.1%	36.4%	91.4%	47.6%	92.6%	91.4%	92.6%
PPV	9.5%	9.5%	23.3%	10.8%	23%	23.3%	23%
NPV	97.3%	98%	94.9%	97.7%	94.7%	94.9%	94.7%
The diagnostic odds ratio (DOR)	3.80	5.16	5.76	5.0	5.33	5.76	5.33

WBC, white blood cells in urine analysis; pyuria: ≥ 5 WBC/mm^3^ in urinalysis; LE, leukocyte esterase; N, nitrite; UTI, urinary tract infection; PPV, positive predictive value; NPV, negative predictive value.

**Table 4.** Factors Associated with Postoperative UTI—Univariate Versus Multivariate Analysis

	Univariate Analysis			Multivariate Analysis		
	OR	95% CI	*P*	OR	95% CI	*P*
Preoperative Double-j stent	2.18	0.85-5.57	.103			
UTI history	4.76	1.87-12.04	.001**	5.03	1.86-13.59	.001**
Preoperative hydronephrosis	0.60	0.24-1.51	.280			
Access sheath use	0.71	0.28-1.77	.467			
Operation time	1.03	1.01-1.05	.025*	1.04	1.01-1.06	.006**
Residual fragment	0.34	0.09-1.17	.086			
WBC in urinanalysis	1.004	1.001-1.007	.002**			
Pyuria	3.80	1.08-13.29	.036			
Leukocyte esterase positivity	5.16	1.17-22.70	.030*			
Nitrite positivity	5.76	2.09-15.86	.001**	8.31	2.61-26.5	.000**

Cox regression analysis.

UTI, urinary tract infection; pyuria: ≥ 5 WBC/mm^3^ in urinalysis; OR, odds ratio.Significant values of univariate analysis should be also bold.*: p < .05, **: p < .01.

## References

[1] Türk C (Chair), Skolarikos A Neisius A Petřík C Seitz KT . Urolithiasis. Netherlands: EAU Guidel Off Arnhem; 2019.

[2] Assimos D Krambeck A Miller NL et al. Surgical management of stones: American Urological Association/Endourological Society Guideline, PART I. J Urol. 2016;196(4):1153 1160. 10.1016/j.juro.2016.05.090) 27238616

[3] Perez Castro E Osther PJS Jinga V et al. Differences in ureteroscopic stone treatment and outcomes for distal, mid-, proximal, or multiple ureteral locations: the Clinical Research Office of the Endourological society ureteroscopy global study. Eur Urol. 2014;66(1):102 109. 10.1016/j.eururo.2014.01.011) 24507782

[4] Geavlete P Georgescu D Niţă G Mirciulescu V Cauni V . Complications of 2735 retrograde semirigid ureteroscopy procedures: a single-center experience. J Endourol. 2006;20(3):179 185. 10.1089/end.2006.20.179) 16548724

[5] Breda A Ogunyemi O Leppert JT Schulam PG . Flexible ureteroscopy and laser lithotripsy for multiple unilateral intrarenal stones. Eur Urol. 2009;55(5):1190 1196. 10.1016/j.eururo.2008.06.019) 18571315

[6] de la Rosette J Denstedt J Geavlete P et al. The Clinical Research Office of the Endourological society ureteroscopy global study: indications, complications, and outcomes in 11,885 patients. J Endourol. 2014;28(2):131 139. 10.1089/end.2013.0436) 24147820

[7] Lo CW Yang SS-D Hsieh CH Chang SJ . Effectiveness of prophylactic antibiotics against post-ureteroscopic lithotripsy infections: systematic review and meta-analysis. Surg Infect (Larchmt). 2015;16(4):415 420. 10.1089/sur.2014.013) 26207401

[8] Kim JW Lee YJ Chung JW et al. Clinical characteristics of postoperative febrile urinary tract infections after ureteroscopic lithotripsy. Investig Clin Urol. 2018;59(5):335-341. 10.4111/icu.2018.59.5.335) PMC6121018 30182079

[9] Senocak C Ozcan C Sahin T et al. Risk factors of infectious complications after flexible uretero-renoscopy with laser lithotripsy. Urol J. 2018;15(4):158 163. 10.22037/uj.v0i0.3967) 29299886

[10] De Coninck V Keller EX Somani B et al. Complications of ureteroscopy: a complete overview. World J Urol. 2020;38(9):2147 2166. 10.1007/s00345-019-03012-1) 31748953

[11] Sohn DW Kim SW Hong CG Yoon BIl Ha US Cho YH . Risk factors of infectious complication after ureteroscopic procedures of the upper urinary tract. J Infect Chemother. 2013;19(6):1102 1108. 10.1007/s10156-013-0632-7) 23783396

[12] Martov A Gravas S Etemadian M et al. Postoperative infection rates in patients with a negative baseline urine culture undergoing ureteroscopic stone removal: a matched case-control analysis on antibiotic prophylaxis from the CROES URS global study. J Endourol. 2015;29(2):171 180. 10.1089/end.2014.0470) 25072350

[13] Southern JB Higgins AM Young AJ et al. Risk factors for postoperative fever and systemic inflammatory response syndrome after ureteroscopy for stone disease. J Endourol. 2019;33(7):516 522. 10.1089/end.2018.0789) 30569755

[14] Moses RA Ghali FM Pais VM Hyams ES . Unplanned hospital return for infection following ureteroscopy—can we identify modifiable risk factors? J Urol. 2016;195(4 Pt 1):931 936. 10.1016/j.juro.2015.09.074) 26410731

[15] Mitsuzuka K Nakano O Takahashi N Satoh M . Identification of factors associated with postoperative febrile urinary tract infection after ureteroscopy for urinary stones. Urolithiasis. 2016;44(3):257 262. 10.1007/s00240-015-0816-y) 26321205

[16] Youssef RF Neisius A Goldsmith ZG et al. Clinical outcomes After ureteroscopic lithotripsy in patients who initially presented with urosepsis: matched pair comparison with elective ureteroscopy. J Endourol. 2014;28(12):1439 1443. 10.1089/end.2014.0343) 25479184

[17] Whiteside SA Razvi H Dave S Reid G Burton JP . The microbiome of the urinary tract - A role beyond infection. Nat Rev Urol. 2015;12(2):81 90. 10.1038/nrurol.2014.361) 25600098

[18] Kohada Y Goriki A Yukihiro K Ohara S Kajiwara M . The risk factors of urinary tract infection after transurethral resection of bladder tumors. World J Urol. 2019;37(12):2715 2719. 10.1007/s00345-019-02737-3) 30915527

[19] Chen D Jiang C Liang X et al. Early and rapid prediction of postoperative infections following percutaneous nephrolithotomy in patients with complex kidney stones. BJU Int. 2019;123(6):1041 1047. 10.1111/bju.14484) 30007112

[20] Fan J Wan S Liu L et al. Predictors for uroseptic shock in patients who undergo minimally invasive percutaneous nephrolithotomy. Urolithiasis. 2017;45(6):573 578. 10.1007/s00240-017-0963-4) 28229195

